# Supplementary material for: Content-rich biological network constructed by mining PubMed abstracts
Source: BMC Bioinformatics. 2004 Oct 8;5:147. doi: 10.1186/1471-2105-5-147 (PMC528731; doi:10.1186/1471-2105-5-147)
Supplement: Additional File 2 — The original results of the above study (non-essential files are deleted to keep the file size under the limit set by BMC bioinformatics). [file 1471-2105-5-147-S2.bz2 › chilibotAdditionalFile2/dip20/391ID10498602E9873/html/NRP1_SEMA3A.html]

 


 **NRP1** and **SEMA3A** 
  
Found 69 abstracts in PubMed, retrieved 20.  
 

 What does Google say? 
 PDF only 
| .edu only 

---

**Interactive relationship** (e.g. stimulation, inhibition, etc)

**Neutral relationship**- Neuropilin 1  [ **NRP1** ]  is the specific receptor for  **Sema3A**  and plays a role in nerve fiber guidance.  Ref: 11830568 Development, 2002
- Cis and trans interactions of L1 with neuropilin 1  [ **NRP1** ]  control axonal responses to semaphorin 3A  [ **SEMA3A** ] .  Ref: 12456642 EMBO J, 2002
- COS 7 cells coexpressing plexin A4 with neuropilin 1  [ **NRP1** ]  were induced to contract by  **Sema3A** , a member of the class 3 semaphorin.  Ref: 12591607 Mech Dev, 2003
- Characterization of neuropilin 1  [ **NRP1** ]  structural features that confer binding to semaphorin 3A  [ **SEMA3A** ]  and vascular endothelial growth factor 165.  Ref: 11886873 J Biol Chem, 2002
- From E7 until E10 NT3 responsive growth cones expressed progressively lower levels of neuropilin 1  [ **NRP1** ] , and were less sensitive to  **Sema3A** .  Ref: 11920727 J Neurobiol, 2002
- Neuropilin 1  [ **NRP1** ]  Npn 1 is a receptor for both semaphorin 3A  [ **SEMA3A** ]   **Sema3A**  and vascular endothelial growth factor 165 VEGF 165.  Ref: 11886873 J Biol Chem, 2002
- Semaphorin 3A  [ **SEMA3A** ]   **Sema3A**  repels growing olfactory axons that express neuropilin 1  [ **NRP1** ]  NP 1, a receptor for  **Sema3A** .  Ref: 12598627 J Neurosci, 2003
- Expression of sema3a1 in somites and neuropilin 1  [ **NRP1** ] , which encodes for a component of the  **Sema3a**  receptor, in angioblasts suggested that Sema3a1 regulates the pathway of the dorsally migrating angioblasts.  Ref: 12783793 Development< MedlineTA>Development, 2003
- Neuropilin 1  [ **NRP1** ]  Npn 1 is a receptor that binds multiple ligands from structurally distinct families, including secreted semaphorins Sema  [ **SEMA3A** ]  and vascular endothelial growth factors VEGF.  Ref: 12852851 Dev Cell, 2003
- VEGF165 and  **Sema3A**  are competitive inhibitors for  **NRP1**  mediated functions in EC and neurons.  Ref: 12613541 Adv Exp Med Biol, 2002

**Non-interactive relationship** (e.g. studied together, co-existance, homology, etc.)

- To assess the role of semaphorin 3A  [ **SEMA3A** ]   **Sema3A**  and its receptor component neuropilin 1  [ **NRP1** ]  Npn 1 in pontocerebellar axon guidance, we compared the distributions of  **Sema3A** , Npn 1, and DiI labeled pontocerebellar axons in neonatal mouse cerebellum.  Ref: 12401447 Mol Cell Neurosci, 2002
- These findings suggest that the neuropilin 1  [ **NRP1** ]  mediated  **Sema3A**  signals regulate arrest and aggregation of sympathetic neuron precursors and sympathetic neurons themselves at defined target sites and axon fasciculation to produce the stereotyped sympathetic nerve pattern.  Ref: 11830568 Development, 2002
- We report that neuropilin 1  [ **NRP1** ]  and  **Sema3A**  mutant mouse embryos, generated by targeted gene disruption, showed displacement of sympathetic neurons and their precursors and abnormal morphogenesis in the sympathetic trunk.  Ref: 11830568 Development, 2002
- After the initial discovery of neuropilin 1  [ **NRP1** ]  as an epitope on axons recognized by a monoclonal antibody, neuropilins were rediscovered in the search for receptors mediating the repulsive actions of class 3 Semaphorins, notably  **Sema3A** .  Ref: 12613540 Adv Exp Med Biol, 2002
- The dermamyotome derived chemorepulsion is exclusively dependent on  **Sema3A**  neuropilin 1  [ **NRP1** ] .  Ref: 12591248 Dev Biol, 2003
- Among its binding partners, L1 immunoglobulin superfamily adhesion molecule Ig CAM associates with neuropilin 1  [ **NRP1** ]  NP 1 to form a semaphorin3A  **Sema3A**  receptor and soluble L1 converts  **Sema3A**  induced axonal repulsion into attraction.  Ref: 12456642 EMBO J, 2002
- Second, L1 and  **NRP1** , the neuropilin responsible for  **Sema3A**  binding, associate through their extracellular domains, forming a cell surface heterocomplex.  Ref: 12613546 Adv Exp Med Biol, 2002
- We also show that  **Sema3A**  suppressed the cell migration activity of sympathetic neurons from wild type but not neuropilin 1  [ **NRP1** ]  mutant embryos in vitro and instead promoted their accumulation into compact cell masses and fasciculation of their neurites.  Ref: 11830568 Development, 2002
- Growth cones extended from E6 DRGs in NT3 containing medium expressed neuropilin 1  [ **NRP1** ]  and collapsed in response to  **Sema3A** .  Ref: 11920727 J Neurobiol, 2002
- This neurotrophin mediated decrease in the collapse response of DRG growth cones was not associated with reduced expression on growth cones of the  **Sema3A**  binding protein neuropilin 1  [ **NRP1** ] .  Ref: 12151545 J Neurosci, 2002
- Temporal regulation of neuropilin 1  [ **NRP1** ]  expression and sensitivity to semaphorin 3A  [ **SEMA3A** ]  in NGF and NT3 responsive chick sensory neurons.  Ref: 11920727 J Neurobiol, 2002
- This study compared the expression of the semaphorin 3A  [ **SEMA3A** ]  receptor subunit, neuropilin 1  [ **NRP1** ] , and the collapse response of growth cones to semaphorin 3A  [ **SEMA3A** ]  for NGF cutaneous and NT3 proprioceptive dependent sensory axons extended from E6 E10 chick embryos.  Ref: 11920727 J Neurobiol, 2002
- Requirement of neuropilin 1  [ **NRP1** ]  mediated  **Sema3A**  signals in patterning of the sympathetic nervous system.  Ref: 11830568 Development, 2002
- On the other hand, growth cones extended from DRGs in NGF containing medium expressed progressively higher levels of neuropilin 1  [ **NRP1** ]  and higher levels of collapse response to  **Sema3A**  over the period from E6 E10.  Ref: 11920727 J Neurobiol, 2002
- Chemorepulsion from the ventral spinal cord does not depend on  **Sema3A**  neuropilin 1  [ **NRP1** ]  but requires axonin 1 SC2 to repel DRG axons.  Ref: 12591248 Dev Biol, 2003
- Pontocerebellar axon guidance neuropilin 1  [ **NRP1** ]  and semaphorin 3A  [ **SEMA3A** ]  sensitivity gradients across basilar pontine nuclei and semaphorin 3A  [ **SEMA3A** ]  variation across cerebellum.  Ref: 12401447 Mol Cell Neurosci, 2002
- Thus, developmental patterning of sensory terminals in the chick spinal cord may arise from changes in both  **Sema3A**  expression in the developing spinal cord and accompanying changes in neuronal expression of the  **Sema3A**  receptor subunit, neuropilin 1  [ **NRP1** ] .  Ref: 11920727 J Neurobiol, 2002
- Using coculture assays composed of tissues derived from the chick embryo or mutant mice treated with function blocking antibodies and phosphatidylinositol specific phospholipase C, we report here that the chemorepellent semaphorin 3A  [ **SEMA3A** ]   **Sema3A**  and its receptor neuropilin 1  [ **NRP1** ]  are required for mediating the dermamyotome and notochord derived, but not the ventral spinal cord derived, chemorepulsive signal for DRG axons.  Ref: 12591248 Dev Biol, 2003
- Neuropilin 1  [ **NRP1** ]  and plexin As form receptor complexes for  **Sema3A**  and plexin As are thought to initiate the intracellular signaling cascade.  Ref: 12559962 Biochem Biophys Res Commun, 2003
